# Supplementary material for: In-situ photomechanical bending in a photosalient Zn-based coordination polymer probed by photocrystallography
Source: Commun Chem. 2025 Nov 28;8:383. doi: 10.1038/s42004-025-01769-8 (PMC12663308; doi:10.1038/s42004-025-01769-8)
Supplement: Supplementary file 2 — Supplementary information [file 42004_2025_1769_MOESM2_ESM.pdf]

In-situ photomechanical bending in a photosalient Zn-based coordination polymer probed by photocrystallography

Samim Khan,[1] Shamim Ahmad,[2] Sanobar Naaz,[1] Niamh T. Hickey,[3,4] Aditya Choudhury,[5] Lauren E. Hatcher,[6] Raghavender Medishetty,[5]* C. Malla Reddy,[2,7]* Sarah Guerin[3,4]* and Mohammad Hedayetullah Mir[1]*

[1] Department of Chemistry, Aliah University, New Town, Kolkata 700156, India. E-mail: chmmir@gmail.com

[2] Department of Chemical Sciences, Indian Institute of Science Education and Research (IISER) Kolkata, Mohanpur, Nadia, West Bengal 741246, India. E-mail: cmallareddy@gmail.com

[3] Department of Chemical Sciences, Bernal Institute, University of Limerick, V94 T9PX, Ireland. E-mail: Sarah.Guerin@ul.ie

[4] SSPC, The Research Ireland Research Centre for Pharmaceuticals, University of Limerick, V94 T9PX, Ireland

[5] Department of Chemistry, IIT Bhilai, Sejbahar, Raipur, Chhattisgarh 492015, India. E-mail: raghavender@iitbhilai.ac.in

[6] School of Chemistry, Cardiff University, Main Building, Park Place, Cardiff CF10 3AT, UK

[7] Department of Chemistry, Indian Institute of Technology Hyderabad, Hyderabad 502284, India

Supplementary information

Supplementary Table S1. Crystal data and refinement parameters of all compounds.

Compounds	1	i$_5$1	i$_{10}$1	i$_{20}$1	1b (Bent)
Formula	$C_{43}H_{32}Cl_2N_2O_5Zn$	$C_{43}H_{31.75}Cl_2N_2O_5Zn$	$C_{42.60}H_{27.70}Cl_2N_2O_{4.60}Zn$	$C_{42.60}H_{27.67}Cl_2N_2O_{4.60}Zn$	$C_{43}H_{32}Cl_2N_2O_5Zn$
Fw	792.97	792.72	777.44	777.40	793.00
Cryst syst	Triclinic	Triclinic	Triclinic	Triclinic	Triclinic
space group	$P\bar{1}$	$P\bar{1}$	$P\bar{1}$	$P\bar{1}$	$P\bar{1}$
a (Å)	7.5425	7.568	7.5660	7.5524	7.4295
b (Å)	13.7619	13.7477	13.734	13.719	13.5061
c (Å)	18.8631	18.906	18.920	18.929	18.8693
α(deg)	102.974	103.066	103.134	103.139	102.881
β(deg)	99.441	99.564	99.466	99.648	99.015
γ (deg)	98.684	98.439	98.420	98.322	97.881
V (Å^3)	1845.87	1854.3	1853.7	1848.5	1793.62
Z	2	2	2	2	2
Temp (K)	294	294	294	294	100
D_{calcd}(g/cm^3)	1.427	1.420	1.393	1.397	1.468
μ(mm^{-1})	0.859	0.856	0.854	0.856	2.734
λ(Å)	0.71073	0.71073	0.71073	0.71073	1.54184
GOF on F^2	1.029	1.024	1.008	0.958	1.036
final R indices $[I > 2\sigma(I)]^{a,b}$	$R1$= 0.0415 $wR2$ = 0.1024	$R1$= 0.0865 $wR2$ = 0.2918	$R1$= 0.1756 $wR2$ = 0.4901	$R1$= 0.1033 $wR2$ = 0.3578	$R1$= 0.0392 $wR2$ = 0.1087

$^aR1 = \Sigma||F_o| - |F_c||/ \Sigma|F_o|$, $^b wR2 = [\Sigma w(F_o^2 - F_c^2)^2/\Sigma w(F_o^2)^2]^{1/2}$

Supplementary Table S2. Selected bond lengths and bond angles of compounds

Bond length (Å)					
	**1**	**$i_{5}1$**	**$i_{10}1$**	**$i_{20}1$**	**1b**
Zn(1)-O(2)	1.9596(19)	1.955(6)	1.955(10)	1.954(8)	1.9650(16)
Zn(1)-O(3)	1.9182(17)	1.919(5)	1.936(9)	1.920(7)	1.9257(16)
Zn(1)-N(1)	2.019(2)	2.026(6)	2.012(8)	2.027(6)	2.016(2)
Zn(1)-N(2)	2.021(2)	2.029(6)	2.039(12)	2.019(10)	2.0108(19)
**Bond angle (°)**					
O(2)-Zn(1)-O(3)	97.99(8)	97.8(2)	96.6(4)	97.0(3)	97.19(7)
O(2)-Zn(1)-N(1)	119.77(8)	119.6(2)	119.7(4)	119.9(3)	118.87(8)
O(2)-Zn(1)-N(2)	101.46(8)	101.2(2)	101.9(4)	101.6(4)	101.70(7)
O(3)-Zn(1)-N(1)	105.03(8)	105.6(2)	105.2(4)	105.2(3)	105.57(8)
O(3)-Zn(1)-N(2)	121.43(9)	121.6(3)	121.6(5)	122.4(4)	121.54(8)
N(1)-Zn(1)-N(2)	111.46(8)	111.3(2)	112.0(4)	111.0(3)	111.97(8)

Supplementary method 1

Photocrystallographic data collection and data processing information

A ground state single crystal X-ray structure was first collected in the dark using a Rigaku Gemini A Ultra dual source diffractometer, equipped with an Atlas S1 CCD X-ray detector and an Oxford Instruments Cryojet-XL liquid nitrogen flow device for temperature control. The data collection, indexing and integration procedures were all performed via the Rigaku sofware CrysAlisPRO, and structures were solved via dual space methods using ShelXT[1] and refined via full matrix least squares on F^2 using ShelXL,[2] both via the Olex2[3] graphical user interface.
The crystal was then irradiated in-situ on the diffractometer under the conditions described in the main manuscript, before a second data collection was again performed in the dark to determine the light-induced structure data.

To first ascertain if any changes had occurred as a result of irradiation, the ground state structure model (atomic coordinates) was first refined against the photoexcited state data as a rigid body, and a Fourier electron density difference map (Photodifference map) generated between the two. New positive residual electron density peaks identified the positions of the new atomic positions that belong to photodimerized excited state. The final mixed GS/ES model for the photoexcited structures was refined as a disorder model using standard PART instructions, with the atomic positions and occupancies of the GS and ES fragments freely refined against an FVAR instruction. This approach allows the ES occupancy to be freely refined from the experimental X-ray data, and these values are quoted in the main manuscript as a percentage with associated estimated standard deviation.

Supplementary Figures

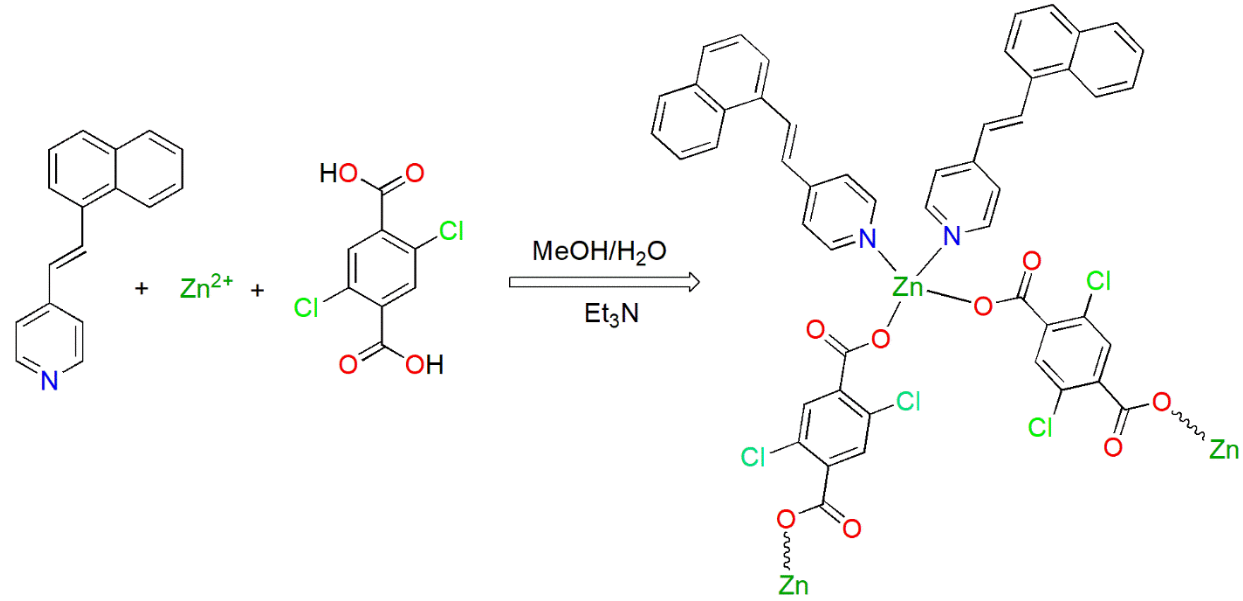

Supplementary Scheme 1: Synthetic route of compound **1**.

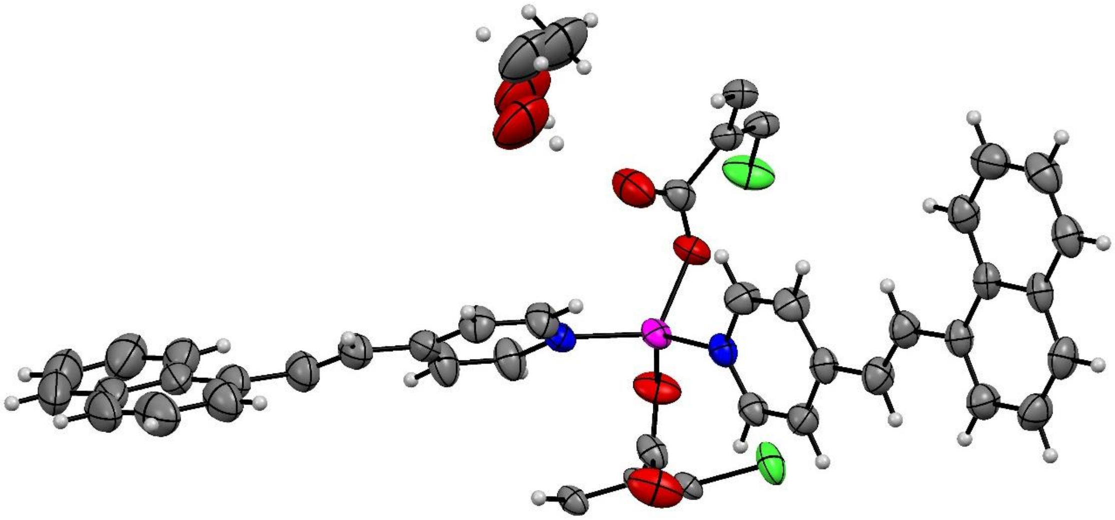

Supplementary Fig. S1: Asymmetric unit of **1** with 50% ellipsoid probability. Carbon: grey; nitrogen: blue; oxygen: red; hydrogen: white; zinc: pink

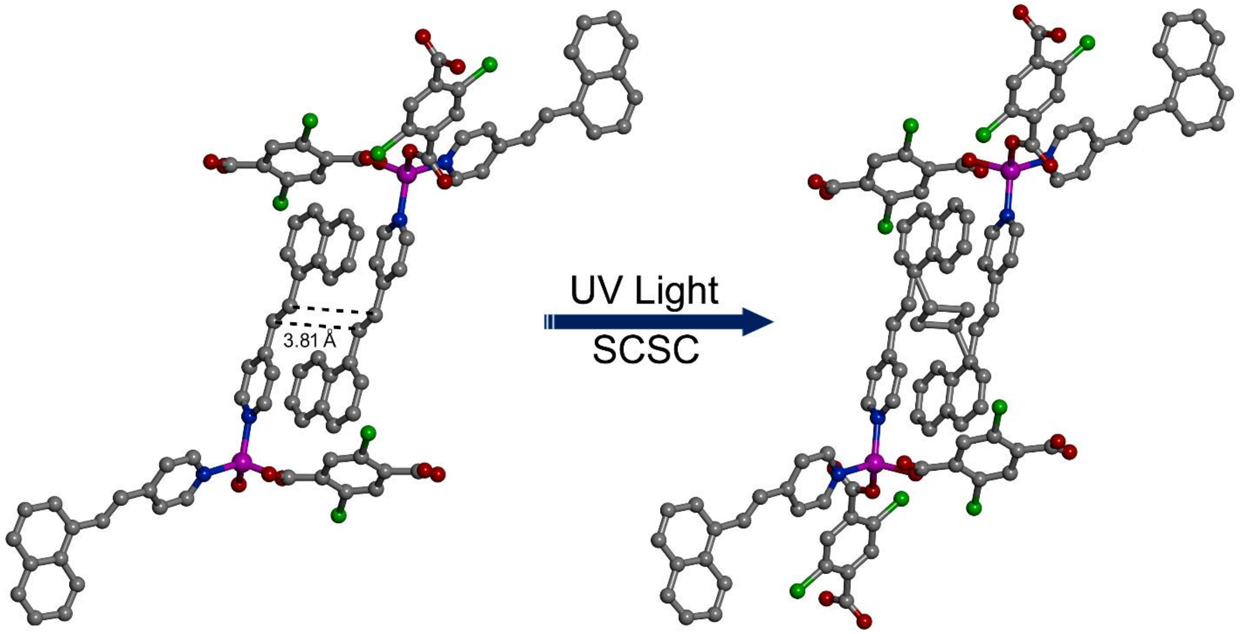

Supplementary Fig. S2: The alignment of 4-nvp ligands between the 1D chains of **1** (left) and partial dimerization of $\mathbf{i_{20}1}$ upon UV irradiation (right).

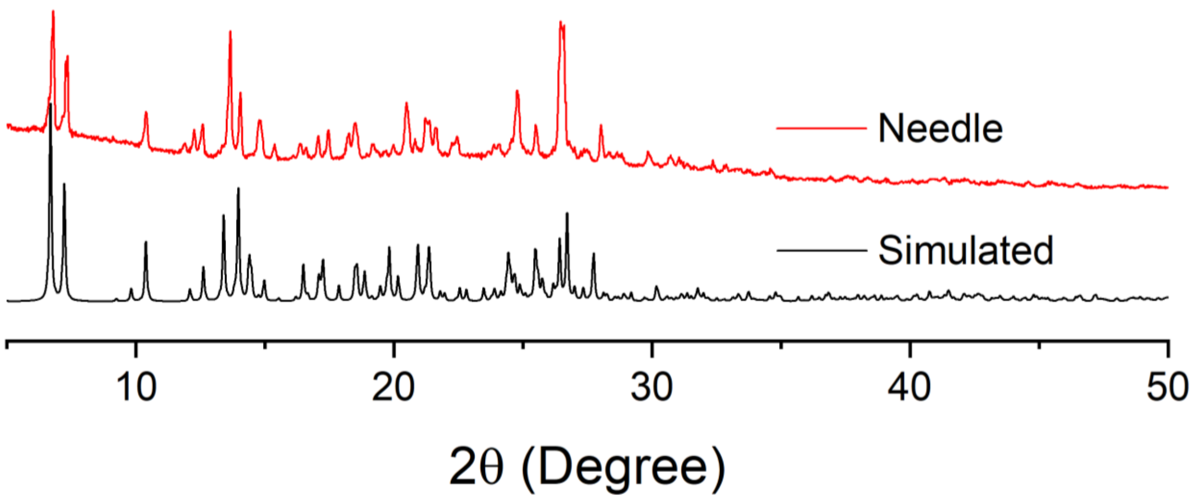

Supplementary Fig. S3: PXRD pattern of needle crystal **1**.

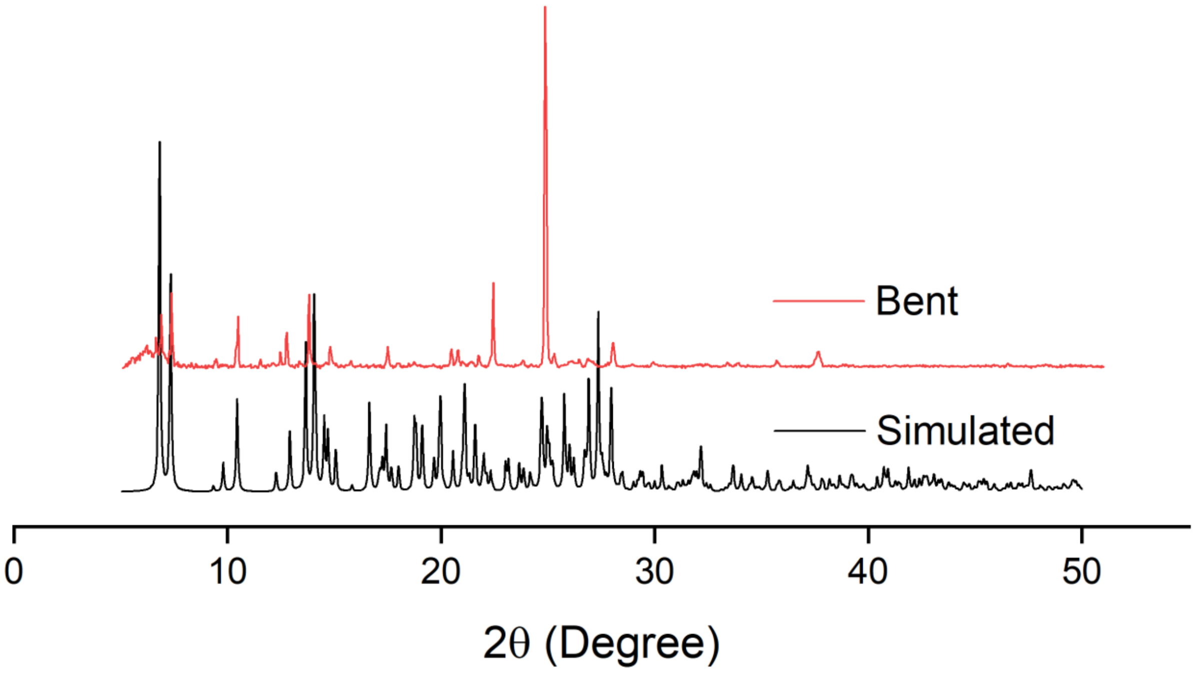

Supplementary Fig. S4: PXRD pattern of bent crystal **1b**.

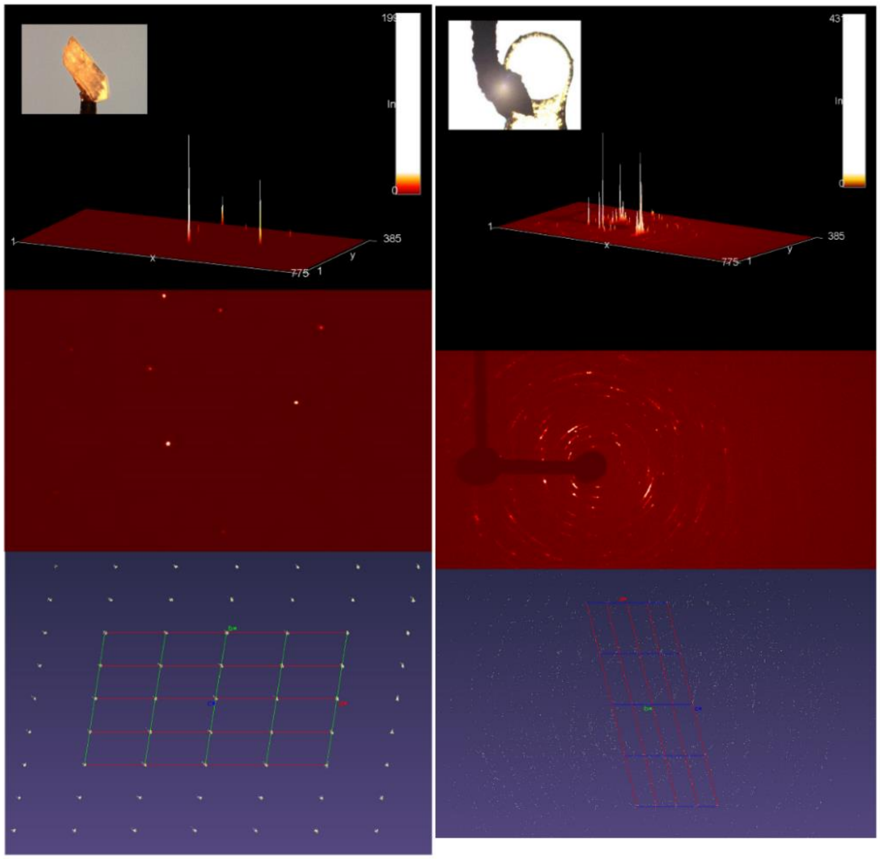

Supplementary Fig. S5: Diffraction images of (a) pristine single crystal and (b) bent region of a highly bent crystal (**1b**)

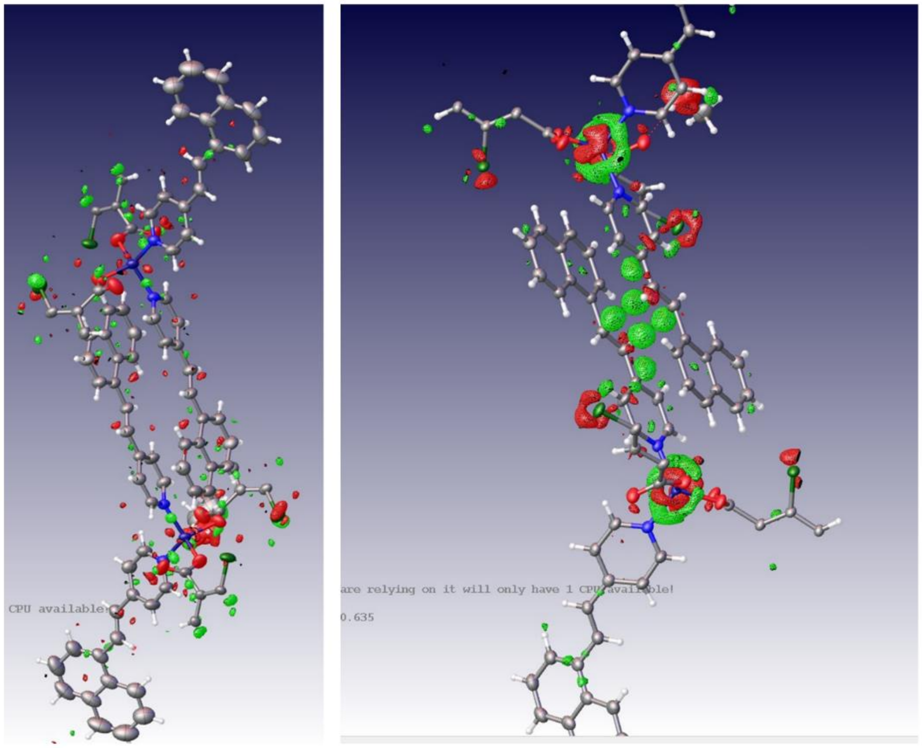

Supplementary Fig. S6: Electron density map of **1** and bent crystal **1b** obtained from Olex2.

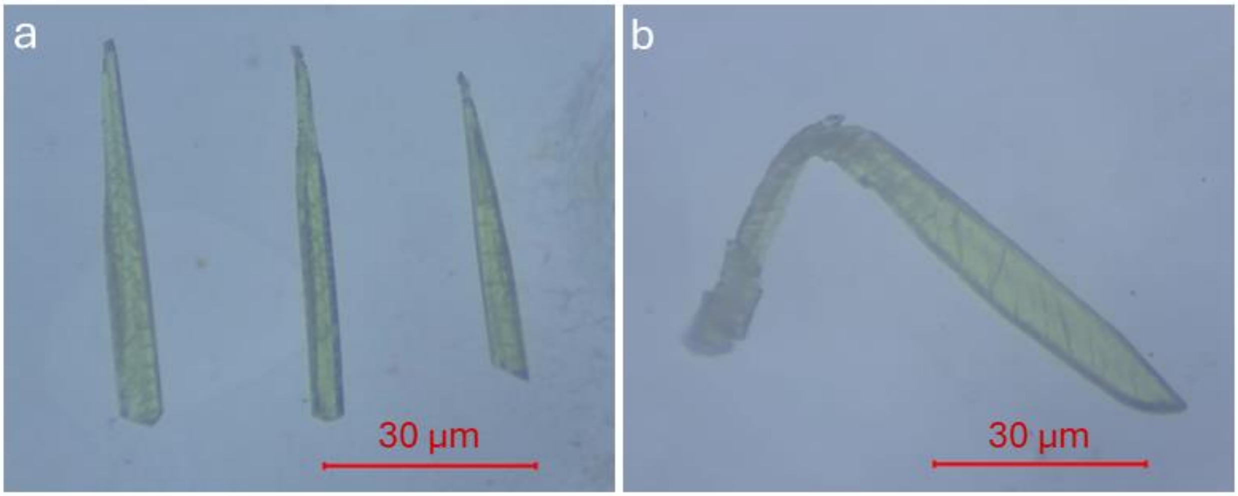

Supplementary Fig. S7: Optical microscopic images of crystals of (a) **1** and (b) **1b**.

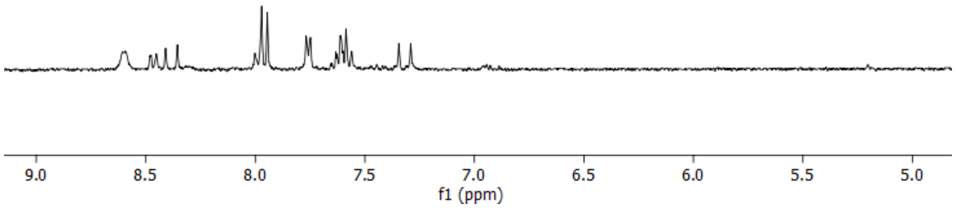

Supplementary Fig. S8: ^{1}H NMR spectrum (400 MHz, DMSO-d_6) of compound **1**.

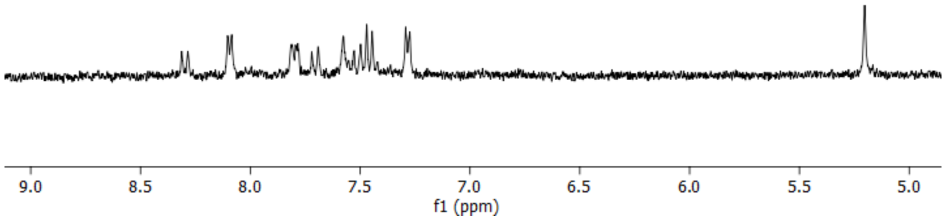

Supplementary Fig. S9: ^{1}H NMR spectrum (400 MHz, DMSO-d_6) of compound **1** after UV irradiation.

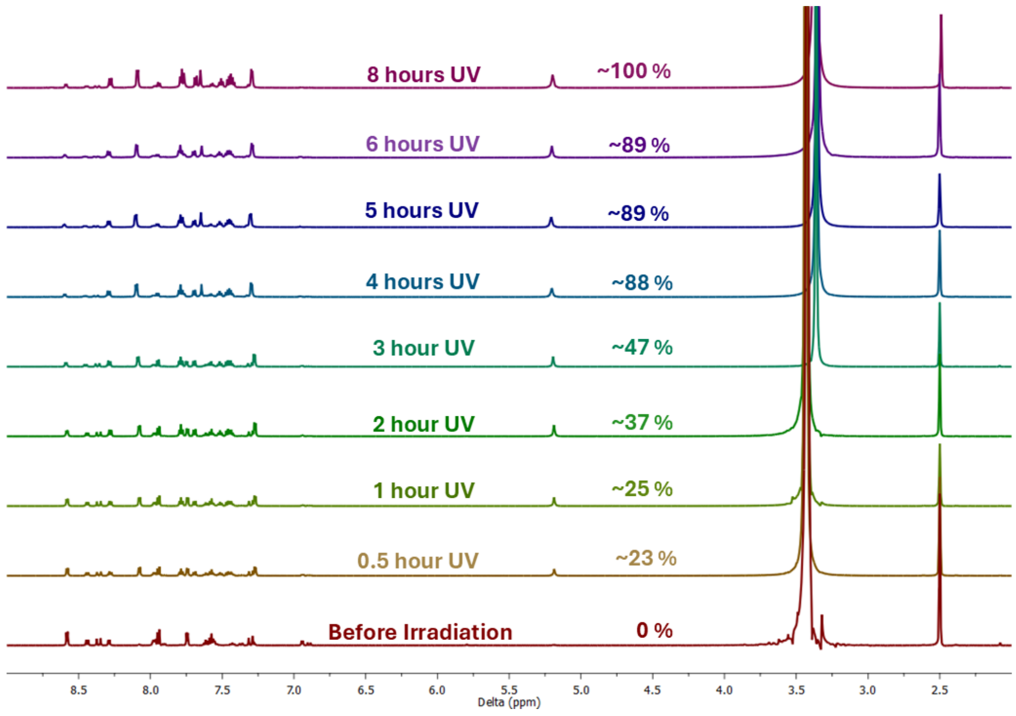

Supplementary Fig. S10: ^{1}H NMR titration of needle crystals of **1**.

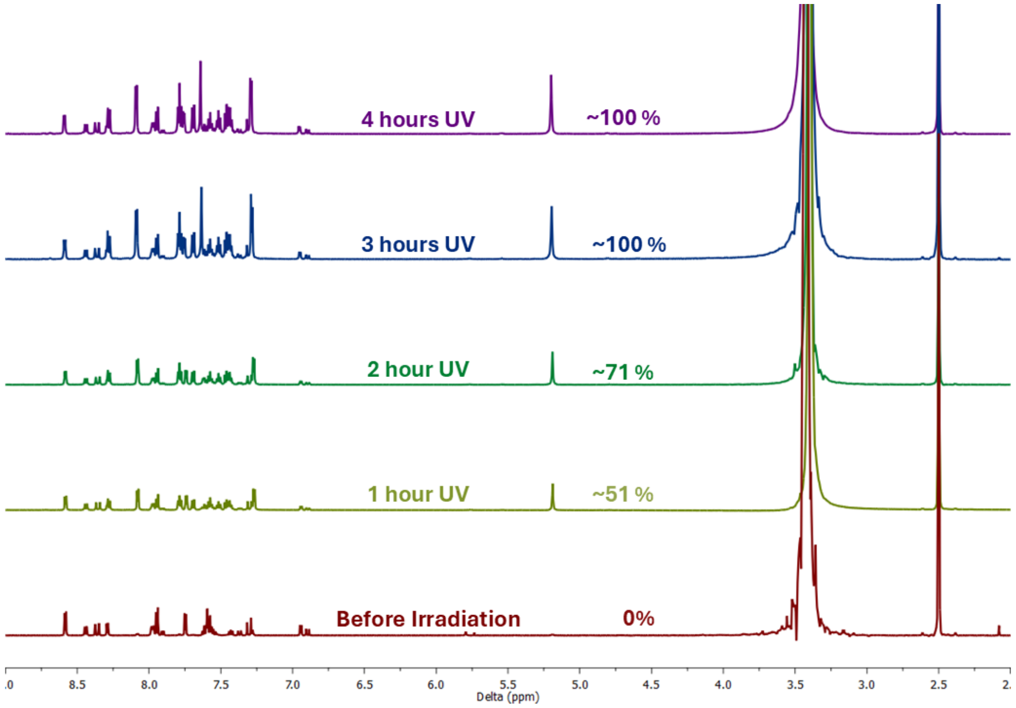

Supplementary Fig. S11: ^{1}H NMR titration of bent crystals (**1b**).

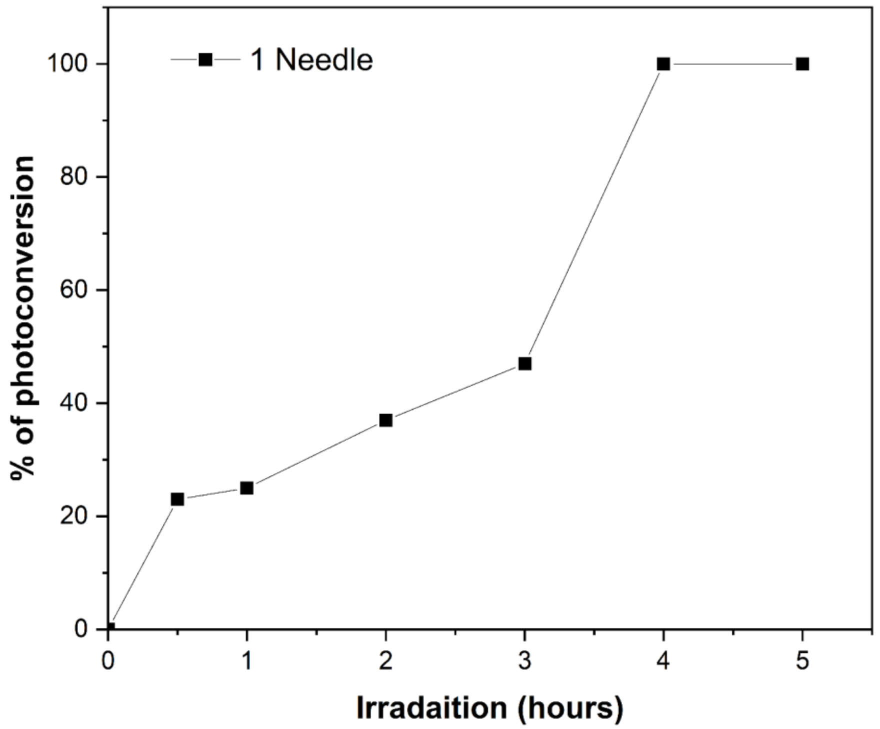

Supplementary Fig. S12: The progress of photoconversion of needle crystals of **1** over various time intervals.

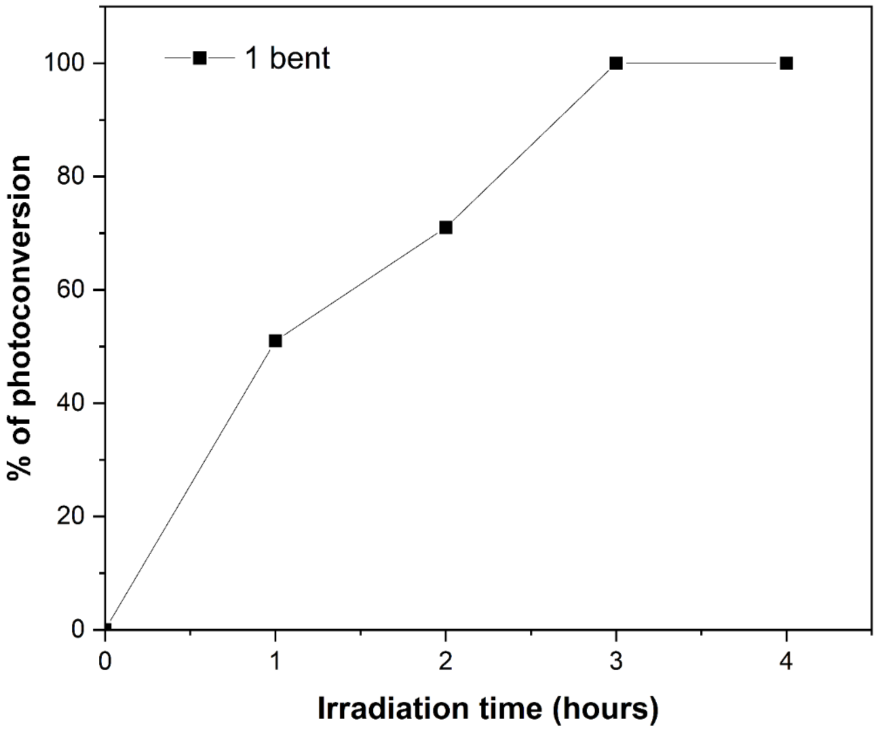

Supplementary Fig. S13: The progress of photoconversion of bent crystals (**1b)** over various time intervals.

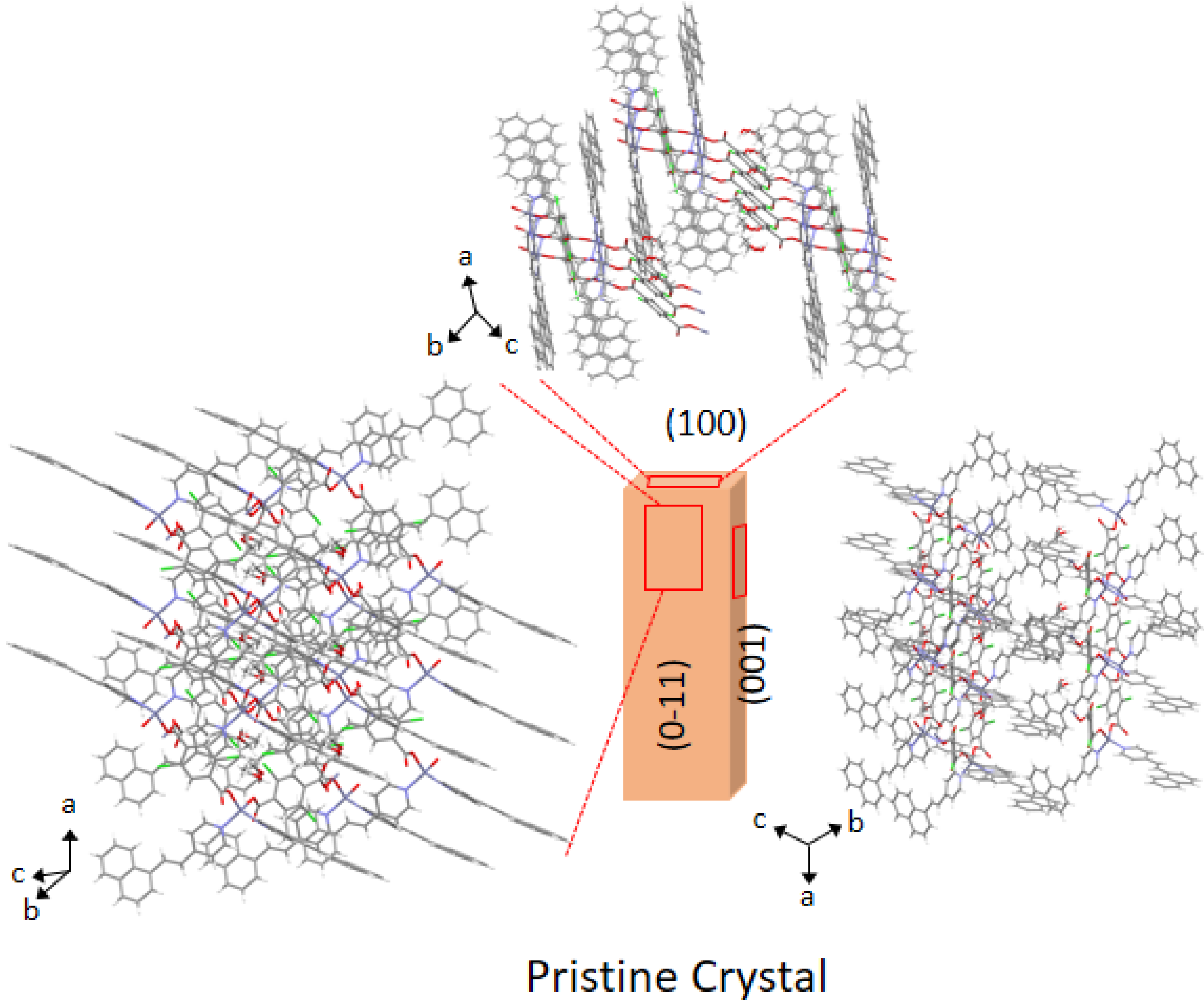

Supplementary Fig. S14: A schematic of the crystal faces and respective packing diagram of crystal **1**.

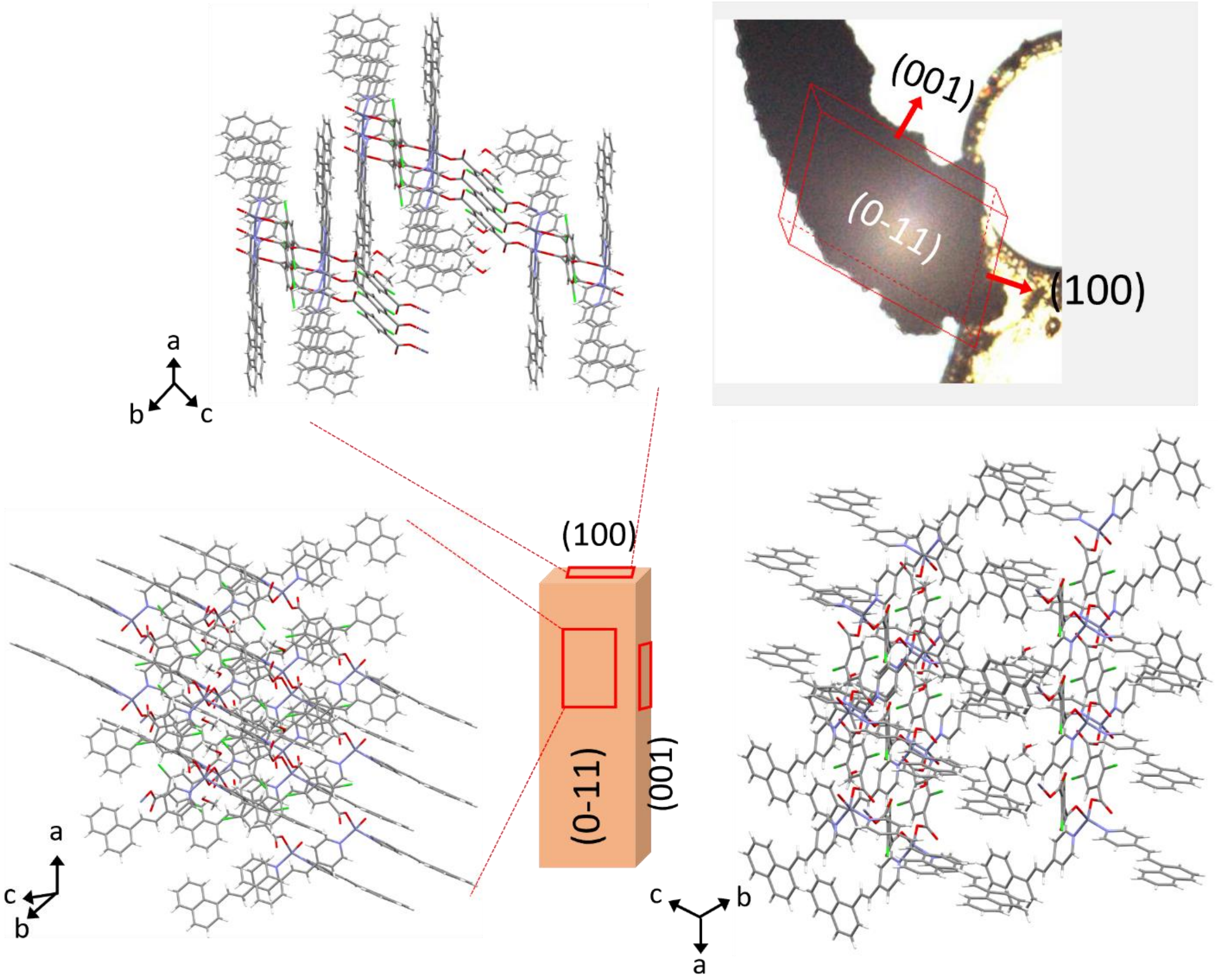

Supplementary Fig. S15: A schematic of the crystal faces and respective packing diagram of crystal **1b**. Face indexing of the bent crystal **1b** is shown in the inset.

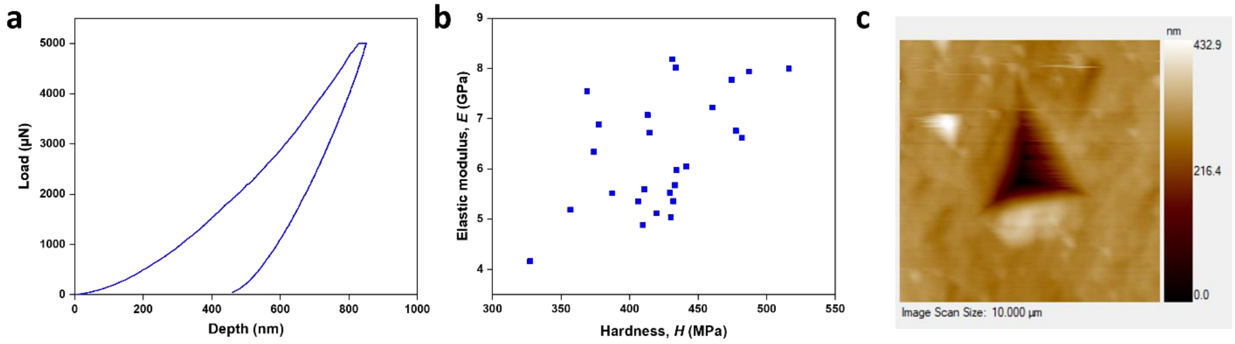

Supplementary Fig. S16: a) Single *P–h* curve, b) scattered plot for elastic modulus vs hardness, c) post indentation 2D SPM image.

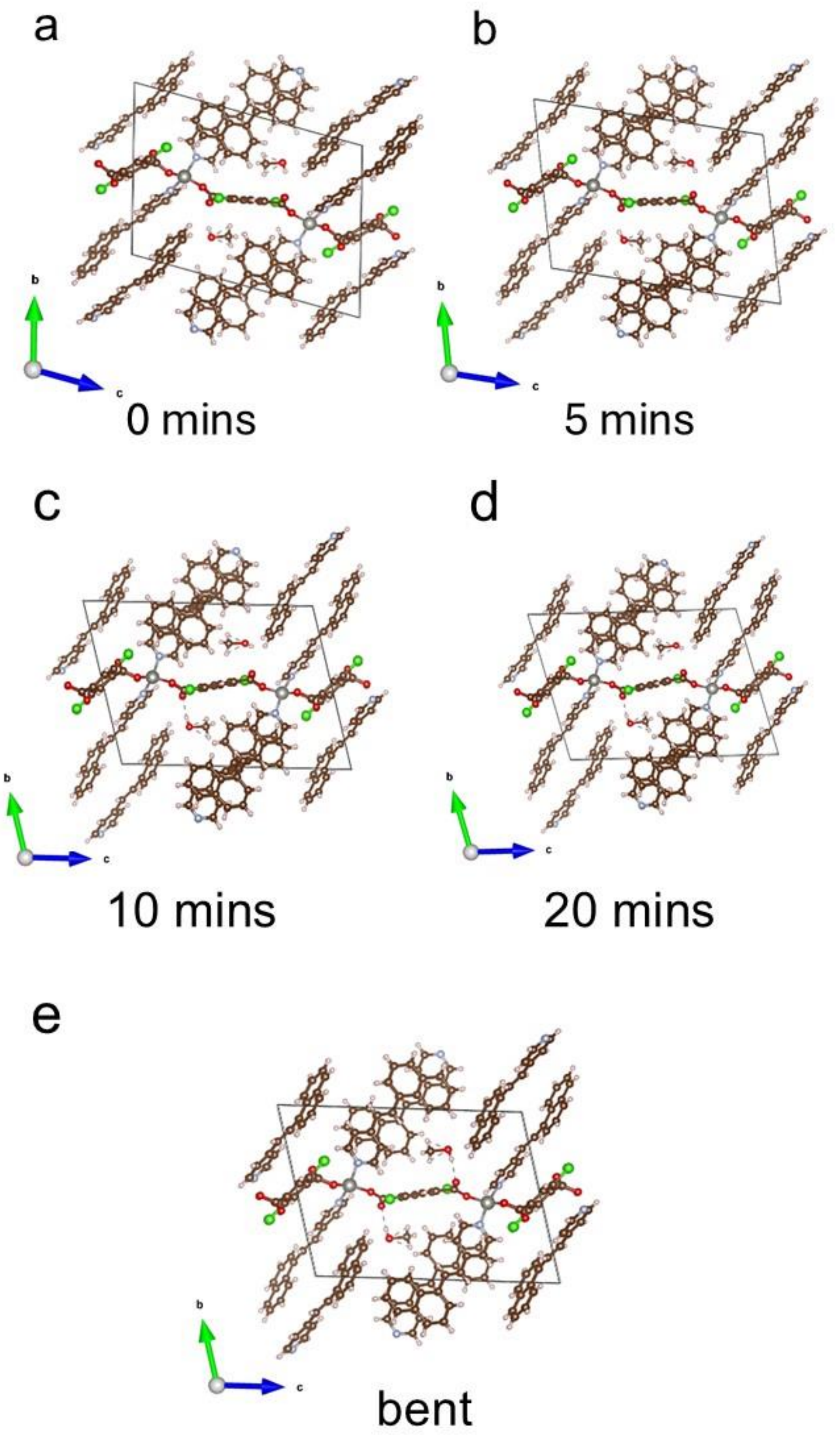

Supplementary Fig. S17: Unit cell of the crystal at different irradiation points a) before irradiation**. b)** 5 minutes of irradiation**. c)** 10 minutes of irradiation. **d)** 20 minutes of irradiation**. e)** after irradiation and bending. Disordered molecules were rebuilt prior to calculation whilst preserving the space group symmetry.

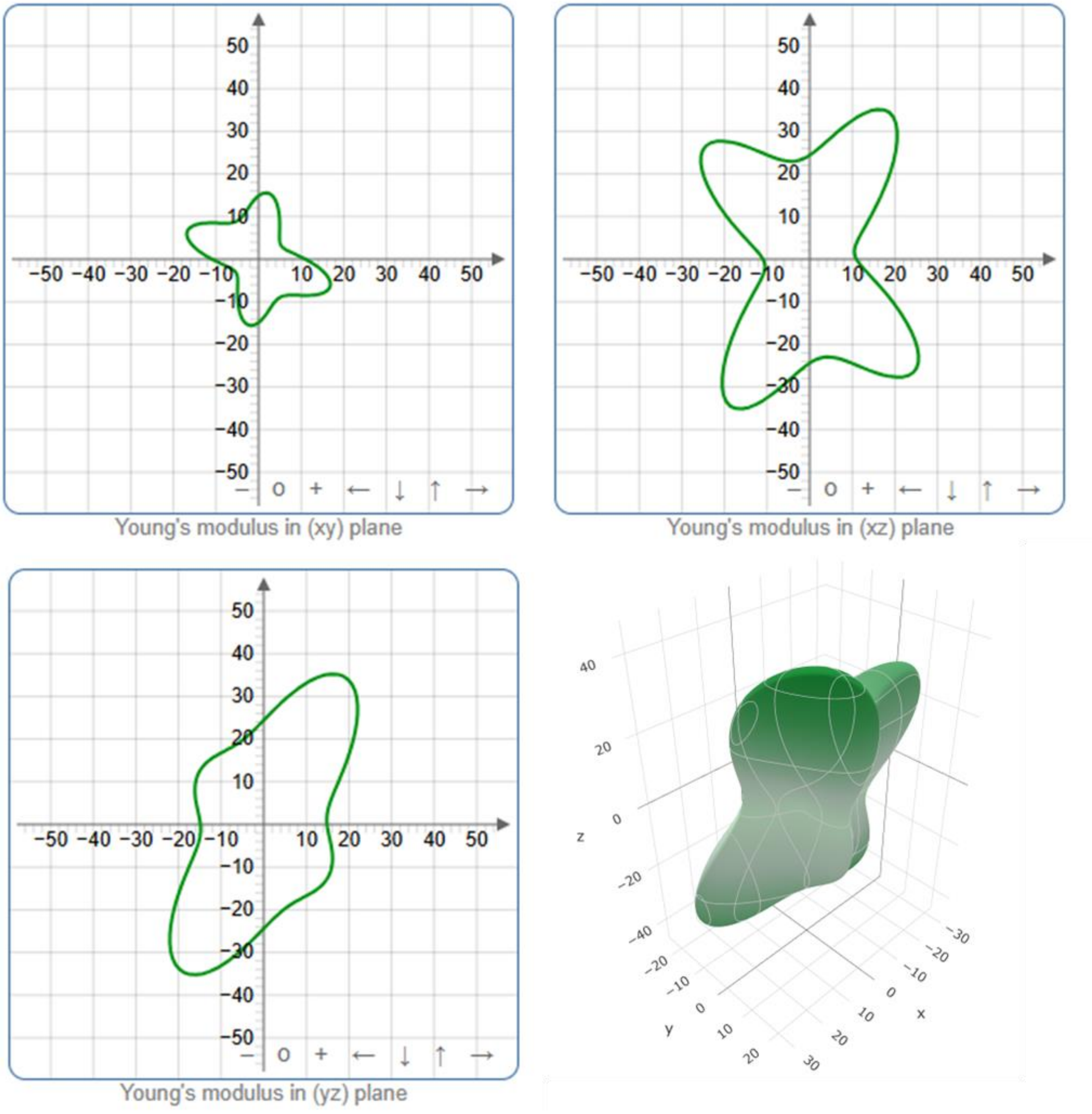

Supplementary Figure S18: 2D and 3D Young's Modulus visualisation of the DFT-optimised pre-irradiated crystal in this study. Derived from the predicted elastic stiffness tensor at https://progs.coudert.name/elate

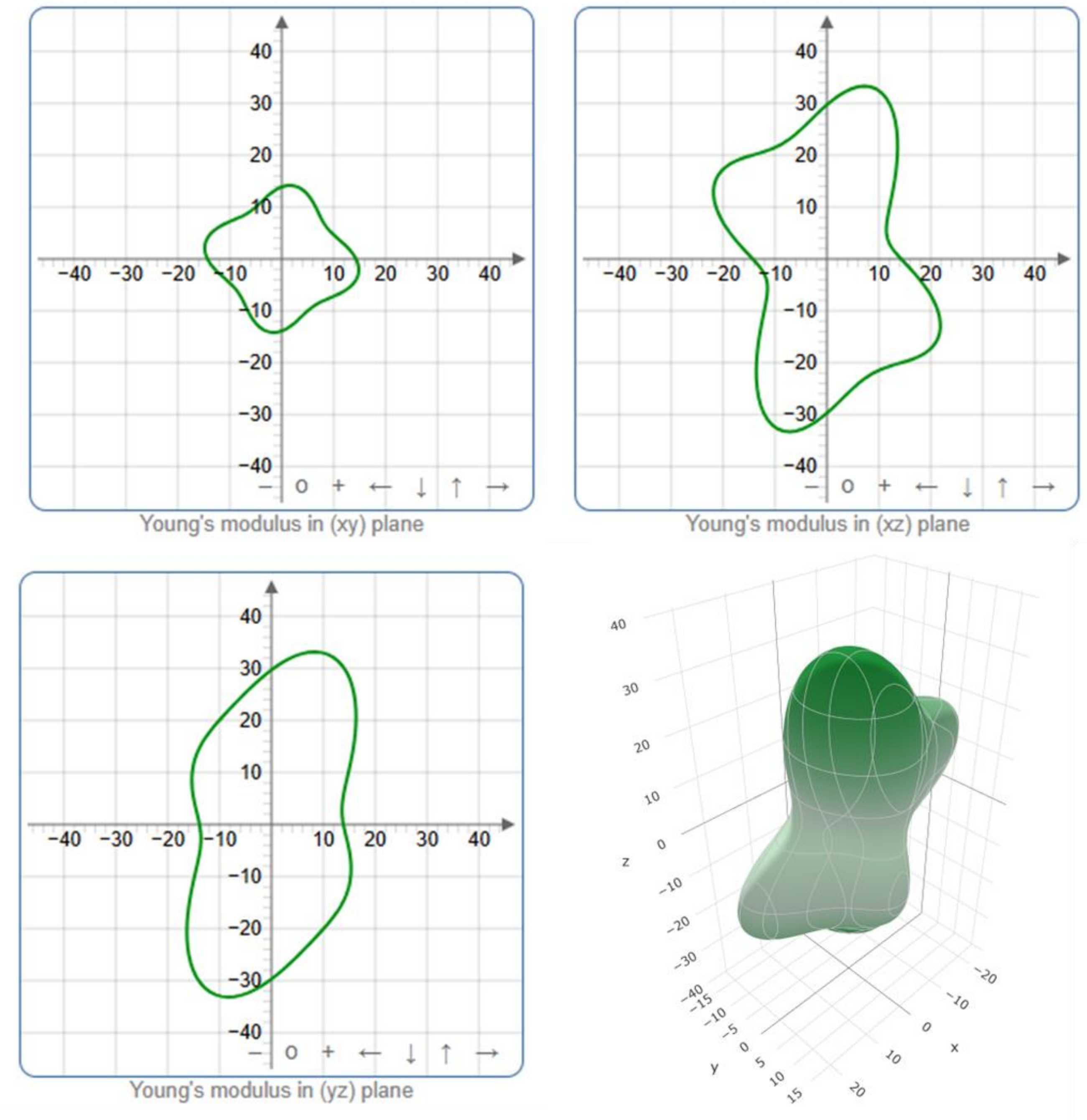

Supplementary Figure S19: 2D and 3D Young's Modulus visualisation for the crystal at 5 minutes of irradiation. Derived from the predicted elastic stiffness tensor at https://progs.coudert.name/elate

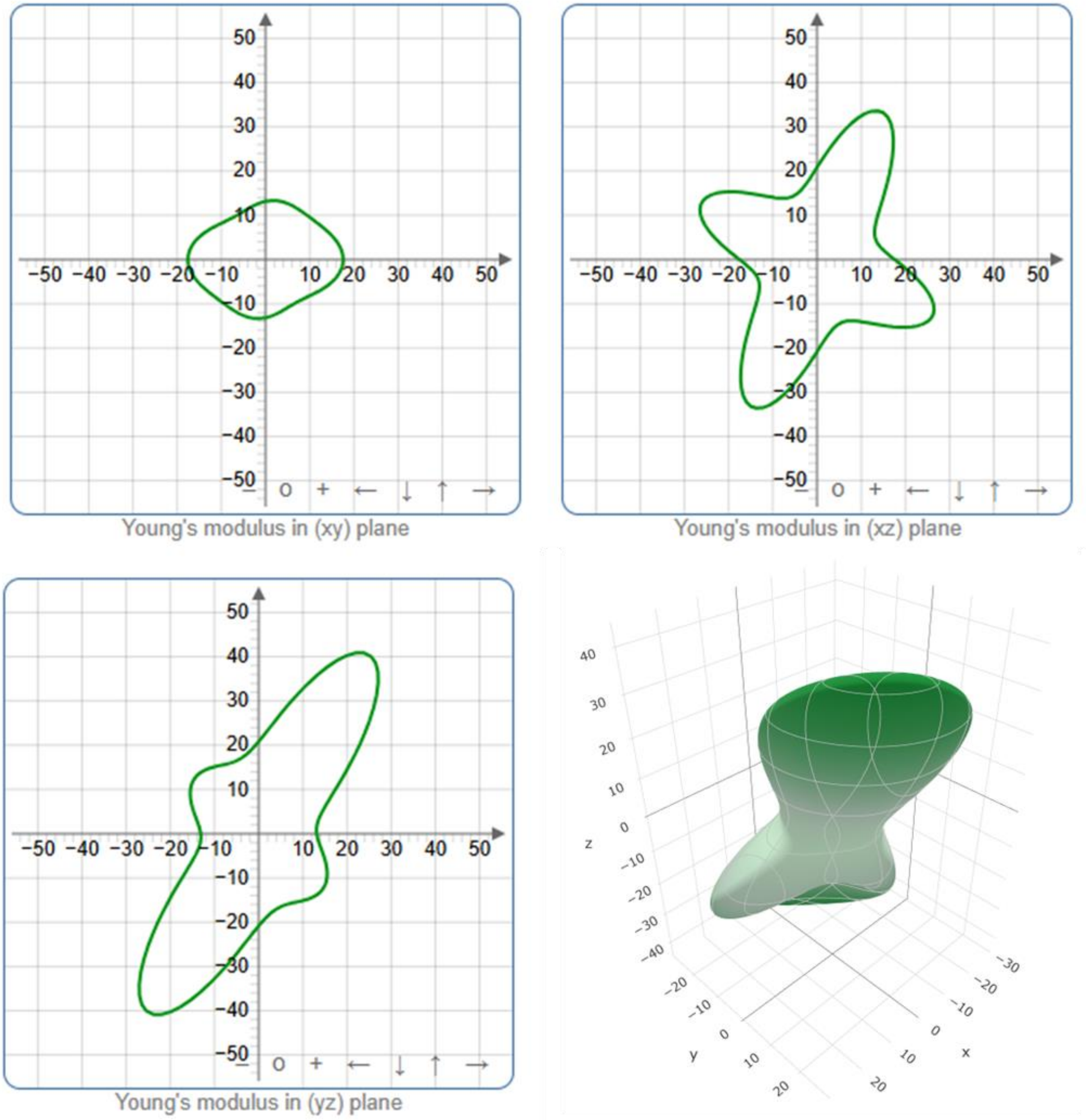

Supplementary Figure S20: 2D and 3D Young's Modulus visualisation for the crystal at 10 minutes of irradiation in this study. Derived from the predicted elastic stiffness tensor at https://progs.coudert.name/elate

Supplementary Table S3: DFT predicted 6x6 elastic stiffness matrices for the pre-irradiated crystal in this study.

	1	2	3	4	5	6
1	24.763	8.365	20.749	2.212	-4.613	-3.819
2	8.365	20.642	16.458	1.756	-0.286	1.016
3	20.749	16.458	54.155	8.941	2.030	0.846
4	2.212	1.756	8.941	12.917	-0.130	-0.232
5	-4.613	-0.286	2.030	-0.130	15.774	1.193
6	-3.819	1.016	0.846	-0.232	1.193	4.252

Supplementary Table S4: DFT predicted 6x6 elastic stiffness matrices for the crystal at 5 minutes of irradiation.

	1	2	3	4	5	6
1	21.649	7.934	13.286	1.546	-5.003	-1.659
2	7.934	19.639	14.704	0.606	-1.894	0.132
3	13.286	14.704	47.229	5.003	1.568	-0.893
4	1.546	0.606	5.003	8.901	1.339	-0.220
5	-5.003	-1.894	1.568	1.339	10.619	0.851
6	-1.659	0.132	-0.893	-0.220	0.851	4.279

Supplementary Table S5: DFT predicted 6x6 elastic stiffness matrix for the crystal at 10 minutes of irradiation.

	1	2	3	4	5	6
1	29.853	12.063	18.901	2.466	-7.426	-2.450
2	12.063	24.583	21.033	4.404	-5.565	-1.654
3	18.901	21.033	52.400	10.817	1.099	-3.265
4	2.466	4.404	10.817	12.691	-1.333	-0.451
5	-7.426	-5.565	1.099	-1.333	12.531	1.818
6	-2.450	-1.654	-3.265	-0.451	1.818	6.154

Supplementary Table S6: DFT predicted 6x6 elastic stiffness matrix for the crystal at 20 minutes of irradiation.

	1	2	3	4	5	6
1	26.990	11.722	18.916	1.784	-6.786	-2.603
2	11.722	24.777	22.901	3.729	-5.704	-1.756
3	18.916	22.901	56.453	9.918	-0.552	-2.767
4	1.784	3.729	9.918	13.557	-1.261	-0.655
5	-6.768	-5.704	-0.552	-1.261	14.150	1.659
6	-2.603	-1.756	-2.767	-0.655	1.659	6.048

Supplementary Table S7: DFT predicted 6x6 elastic stiffness matrix for the crystal post-irradiation.

	1	2	3	4	5	6
1	19.492	4.190	16.865	-1.048	-8.987	-2.859
2	4.190	10.915	10.985	-3.763	-1.264	0.634
3	16.865	10.985	48.158	-1.672	-1.182	-0.306
4	-1.084	-3.763	-1.672	8.552	1.261	-0.343
5	-8.987	-1.264	-1.182	1.261	14.488	-1.448
6	-2.859	0.634	-0.306	-0.343	-1.448	3.174

Supplementary References

(1) G. Sheldrick, M.SHELXT–Integrated space group and crystal structure determination. *Acta Crystallogr. A,* 2015, **71**, 3–8.

(2) G. Sheldrick, *Acta Crystallographica Section C* **2015**, *71*, 3-8.

(3) O. V. Dolomanov, L. J. Bourhis, R. J. Gildea, J. A. K. Howard, H. Puschmann, Journal of Applied Crystallography 2009, 42, 339-341.
