# Supplementary material for: In-situ photomechanical bending in a photosalient Zn-based coordination polymer probed by photocrystallography
Source: Commun Chem. 2025 Nov 28;8:383. doi: 10.1038/s42004-025-01769-8 (PMC12663308; doi:10.1038/s42004-025-01769-8)
Supplement: Supplementary file 3 — Description of Additional Supplementary Files [file 42004_2025_1769_MOESM3_ESM.docx]

**Description of Additional Supplementary Files**

**File name**: Supplementary Data 1

**Description**: Crystallographic data in CIF format for **1**

**File name**: Supplementary Data 2

**Description**: Crystallographic data in CIF format for **i_5_1**

**File name**: Supplementary Data 3

**Description**: Crystallographic data in CIF format for **i_10_1**

**File name**: Supplementary Data 4

**Description**: Crystallographic data in CIF format for **i_20_1**

**File name**: Supplementary Data 5

**Description**: Crystallographic data in CIF format for **1b**

**File name**: Supplementary Movies 1-4

**Description**: Violent mechanical motion (cracking, bending and jumping) of Zn CP crystals under UV light
